# Dietary supplementation of oregano essential oil, lauric acid, and a multi-enzyme complex enhances productivity, metabolic status, and gut health in Dagu breeder hens

**DOI:** 10.1093/jas/skaf337

**Published:** 2025-10-21

**Authors:** Xiaotong Li, Changmin Jin, Huiying Li, Qiyue Zhang, Libo Zhang, Lizhi Jin, Caimei Shao, Shuang Ren, Ying Yu, Weijie Feng, Hui Yang, Shimeng Huang, Donghui Shi

**Affiliations:** Department of Animal Husbandry & Veterinary Medicine, Jinzhou Medical University, Jinzhou, 121001, P.R. China; Liaoning Provincial Key Laboratory of Animal Product Quality and Safety, Jinzhou Medical University, Jinzhou, 121001, P.R. China; Department of Animal Husbandry & Veterinary Medicine, Jinzhou Medical University, Jinzhou, 121001, P.R. China; Liaoning Provincial Key Laboratory of Animal Product Quality and Safety, Jinzhou Medical University, Jinzhou, 121001, P.R. China; Department of Animal Husbandry & Veterinary Medicine, Jinzhou Medical University, Jinzhou, 121001, P.R. China; Liaoning Provincial Key Laboratory of Animal Product Quality and Safety, Jinzhou Medical University, Jinzhou, 121001, P.R. China; Department of Animal Husbandry & Veterinary Medicine, Jinzhou Medical University, Jinzhou, 121001, P.R. China; Liaoning Provincial Key Laboratory of Animal Product Quality and Safety, Jinzhou Medical University, Jinzhou, 121001, P.R. China; Department of Animal Husbandry & Veterinary Medicine, Jinzhou Medical University, Jinzhou, 121001, P.R. China; Liaoning Provincial Key Laboratory of Animal Product Quality and Safety, Jinzhou Medical University, Jinzhou, 121001, P.R. China; Guangzhou Meritech Bioengineering Co. Ltd., Guangzhou, 510300, P.R. China; Wellhope Foods Co. Ltd., Shenyang, Liaoning Province 110121, P. R. China; Wellhope Foods Co. Ltd., Shenyang, Liaoning Province 110121, P. R. China; Wellhope Foods Co. Ltd., Shenyang, Liaoning Province 110121, P. R. China; Wellhope Foods Co. Ltd., Shenyang, Liaoning Province 110121, P. R. China; Fujian Vocational College of Agriculture and Industry-Education Integration Center, Fuzhou, Fujian, 350303, China; National Key Laboratory of Livestock and Poultry Nutrition and Feeding, College of Animal Science and Technology, China Agricultural University, Beijing, 100193, China; Department of Animal Husbandry & Veterinary Medicine, Jinzhou Medical University, Jinzhou, 121001, P.R. China; Liaoning Provincial Key Laboratory of Animal Product Quality and Safety, Jinzhou Medical University, Jinzhou, 121001, P.R. China

**Keywords:** complex enzyme preparation, laying performance, nutrient apparent metabolism, oregano essential oil, lauric acid

## Abstract

Phytogenic compounds and enzymes represent promising strategies to modulate gut microbiota and improve nutrient absorption in poultry. The study was conducted to investigate the effects of oregano essential oil and lauric acid (OEA) as well as complex enzyme preparation (CEP) on laying performance, egg quality, nutrient apparent metabolism, and intestinal morphology of Dagu breeder hens. A total of 288 38-wk-old Dagu breeder hens with similar body weight and condition procured, were randomly divided into 4 groups with 6 replicates of 12 hens. Diet feed additives supplement strategies were as follows: a basal diet as control (CON), while the experimental treatment groups received basal diet containing 200 mg/kg of OEA (LCO), 200 mg/kg of CEP (LCE), and 200 mg/kg of OEA + CEP (LOE). The experiment lasted 40 d. Data were analyzed by one-way ANOVA followed by Duncan’s multiple range test using SPSS software, with statistical significance declared at *P *< 0.05. The results showed that OEA + CEP supplementation significantly increased the laying rate to 69.56%, improved the feed-egg ratio to 3.31, and increased the monthly total egg weight to 18.86 kg/bird, and the OEA supplementation had significant reduced the broken-soft egg ratio by 85.19% than the CON group (*P *< 0.05). Additionally, dietary supplementation with OEA and OEA + CEP increased eggshell strength (7.66%) and Haugh unit (2.01%) level (*P *< 0.05). Notably, OEA + CEP significantly improved the antioxidant performance of total antioxidant capacity (7.02%), glutathione peroxidase (11.31%), total superoxide dismutase (5.02%), and malondialdehyde (7.93%) on Dagu breeder hens (*P *< 0.05), and OEA supplemental in diet significantly increased the level of total antioxidant capacity (6.66%) and glutathione peroxidase (7.97%). Compared to the CON group, the apparent metabolizable energy (4.33%), dry matter (4.15%), crude protein (94.92%), calcium (4.83%), and phosphorus (2.53%) levels in the treatment groups significantly increased (*P *< 0.05). The dietary supplementation with OEA +CEP improved the α-amylase (9.23%), lipase (12.86%), and trypsin (9.99%) activities and the villus to crypt ratio in jejunum and ileum (*P *< 0.05). These findings characterize that the dietary treatment with OEA and CEP alone or in combination improves production performance and health status in Dagu breeder hens. Furthermore, combination OEA and CEP demonstrates the potential value to poultry industry.

## Teaser

Text 1: This study demonstrates that combining oregano essential oil/lauric acid (OEA) and compound enzymes (CEP) enhances egg production, quality, and overall health in Dagu breeder hens, offering a natural, antibiotic-free strategy for sustainable poultry farming.

Text 2: The synergistic use of OEA and CEP improves antioxidant capacity, immunity, and digestion in Dagu hens, providing an eco-friendly nutritional solution to boost productivity while reducing reliance on antibiotics in poultry production.

## Introduction

The Dagu chicken, obtaining the alias of Zhuanghe Dagu chicken, which is a native breed with dual purposes (Qin et al., 2015), mainly reared in Zhuanghe area of Dalian City (Liaoning Province, China). It was included in the ‘China Poultry Breeds Record’. The breed is characterized by its large size, strong adaptability, and good disease resistance (Gu and Li, 2020). Its egg is known for its large weight and nutritional value, being rich in trace elements and vitamins, and also contains lecithin, which has a beneficial effect on brain health and intelligence. Furthermore, it is also a good poultry product with delicious meat and delicate flavor (Zhu et al., 2024). Hereby, the dual-purpose chicken has attracted increasing attention, highlighting the need for further studies on their nutritional systems to promote sustainable development of the industry.

A plant extract, essential oil (EO), as a feed additive has been widely concerned by the piglets and poultry industry (Zeng et al., 2015; Gao et al., 2022). Oregano (*Origanum vulgare* L.) is a perennial herb of the Labiatae family, widely used as a medicinal, spice, and feed additives (Ünal et al., 2014). Oregano essential oil (OEO) shows extensive antibacterial activity with good inhibitory effects on *Escherichia coli* and *Staphylococcus aureus* (Hao et al., 2022). The main components (thymol and carvacrol) are responsible for the characteristic odor, antimicrobial and antioxidant activity (Rodriguez-Garcia et al., 2016; Falleh et al., 2020). Studies have shown that OEO can enhance the integrity of intestinal barrier and regulate intestinal flora (Kim et al., 2023), and improve body weight gain and feed conversion in laying hens and broiler chickens (Eler et al., 2019). Lauric acid (LA) is a saturated fatty acid with 12 carbon atoms, which is recognized as a promising healthy fatty acid in animal husbandry (Zhan et al., 2024). It plays an important role in disease resistance and improving immune physiological function (Zhang et al., 2009; Zheng et al., 2022). Moreover, previous studies have shown LA reduces deoxynivalenol-induced damage in intestinal porcine epithelial cell line (Kim and Lee, 2024). Notably, mixed essential oils and lauric acid (OEA) not only showed synergistic beneficial effects on growth performance and gut health (Choi et al., 2022), but exhibited positive function in lipase activity in jejunum, immune status by increasing serum antibodies and anti-inflammation function (Jiang et al., 2024).

In parallel, exogenous enzyme preparations are widely used in poultry diets to mitigate anti-nutritional factors (e.g., NSP and phytate), enhance nutrient digestibility, and improve overall production performance (Roberts and Choct, 2006; Ravindran, 2013). Studies demonstrate that enzymes—both single and complex formulations—can increase egg production (Flores-Cervantes et al., 2011), improve feed conversion (Mathlouthi et al., 2003), and increase intestinal villi height (Westbrook and Cherian, 2019) in poultry and swine. Complex enzymes containing carbohydrases like xylanase, cellulase, and β-glucanase are particularly effective in breaking down dietary fiber and improving nutrient availability.

Given their complementary mechanisms—OEA promoting gut health and CEP enhancing nutrient availability—we hypothesized that their combination would yield synergistic effects. While our previous research indicated positive results in broilers, their efficacy in laying hens remains unexplored. Our previous study has found that the dietary supplementation with 200 mg/kg OEA and CEP had a potential positive effect on the performance of broilers. Therefore, the purpose of this study was to whether dietary supplementation with 200 mg/kg OEA and CEP alone or combination could improve the laying performance, egg quality, serum parameters, apparent metabolism, and intestinal morphology of Dagu breeder hens.

## Materials and Methods

The animal protocols for this study were approved by the Animal Care and Use Committee of Jinzhou Medical University.

### Birds, experimental design, housing, and diet

A total of 288 38-wk-old Dagu breeder hens with similar body weight and condition procured, which were obtained from Liaoning Zhuanghe Dagu Chicken Original Breeding Farm Co., were randomly divided into 4 groups with 6 replicates of 12 hens. Diet feed additives supplement strategies were as follows: a basal diet as control (CON), while the experimental treatment groups received basal diet containing 200 mg/kg of OEA (LCO), 200 mg/kg of CEP (LCE), and 200 mg/kg of OEA and CEP (LOE). The inclusion level for the combination group was set at 100 mg/kg for OEA and 100 mg/kg for CEP to investigate whether a lower total dosage of the combined additives could elicit a synergistic effect comparable to or greater than the higher dosage of each additive alone. The experiment lasted 40 d, including a 7d acclimation period and 33d formal experimental period. All hens used in this study were individually housed in single cages. No dead or weak hens were found during the acclimation period. After 1 week of the adaptation, the hens were selected at symmetrical positions in the barn for grouping, weighing and measuring laying performance. All hens were provided ad libitum access to fresh water.

Diet supplementation additives OEA was provided by Meritech Bioengineering Co. Ltd (Guangzhou, China), which is a novel commercial blend of OEO and lauric acid (commercial name: MeriJoy). OEO consists vof more than 30 substances in which both carvacrol and thymol are more than 70% in total. The enzyme preparations used in this experiment were all feed-grade, and the concentrations of combined protease, amylase, combined xylanase, combined mannase, and cellulase in the complex enzyme preparation (CEP) were mixed at ≥4,000 μ/g, ≥600 μ/g, ≥18,500 μ/g, ≥10,000 μ/g, and ≥200 μ/g, respectively. Powdered, added in a solid form in proportion. The basal diets were formulated with corn-soybean meal, according to China’s Feeding standard of chicken (2004) and Technical Regulations on Feed Nutrition for Large Boned Chickens, while trace elements and vitamin additions were formulated according to the NRC (1994) recommendations. The composition and nutrient levels of the basal diet are listed in Table 1. All hens remained in good health and medical intervention was not applied to any birds during the whole feeding period.

**Table 1.** Composition and nutrient levels of the basal diets

Items	Composition
**Ingredients**	**Content**
Corn	58.50
Soybean meal	14.46
DDGS	9.80
Corn germ meal	3.00
Soybean oil	0.81
Feather powder	2.00
Calcium biphosphate	0.72
Fine stone powder	8.94
Zeolite	0.30
Limestone	0.20
Sodium chloride	0.24
Baking soda	0.06
L-lysine (70%)	0.17
DL-methionine (99%)	0.14
L-threonine (98.5%)	0.02
Tryptophan	0.02
Isoleucine	0.03
Choline chloride (50%)	0.09
Premix^1^	0.50
Total	100.00
Nutrient levels^2^	
Metabolizable energy (MJ/kg)	11.09
Crude protein	15.90
Crude fiber	3.51
Crude fat	2.91
Ca	3.97
Total phosphorus	0.53
L-lysine	0.80
DL-methionine	0.51
Met +Cys	0.79
L-threonine	0.76

1 Premix provided the following per kg of diet: Fe (as ferrous sulfate) 70 mg, Cu (as copper sulfate) 20 mg, Zn (as zinc sulfate)70 mg, Se (as sodium selenite) 0.5 mg, VA 7 000 IU, VD3 2 500 IU, VE 30 mg, VK31 mg, VB_1_ 1.5 mg, VB_2_ 4 mg, VB_6_ 1.5 mg, nicotinic acid 30 mg, folic acid 0.55 mg, D-pantothenic acid10 mg, VB_12_ 0.02 mg, biotin 0.16 mg.

2 ME, CP, CF, EE, Ca, and TP were measured values, while the others were calculated values.

### Sample collection

On day 30 of the trial, two hens with no significant difference in average body weight were randomly selected from each replicate. After fasting (water supply) for 12 h, hens were weighed. Blood samples were collected from the wing vein and centrifuged at 3,500 rpm × 20 min to separate sera. The supernatant was taken and packaged in an EP tube then stored at −20°C to be used for analysis of serum parameters. Following the blood sampling procedure, the birds were euthanized by exsanguination and necropsied. Approximately 1.5 cm proximal sections of the jejunum and ileum were excised and rinsed with ice-cold phosphate-buffered saline, then fixed in 4% paraformaldehyde for 24 h and rinsed with running water for 24 h for intestinal histology. Mucosa from the jejunum and ileum was collected and stored at −80°C for digestive enzyme assays. 6 eggs were randomly selected from each replicate and stored at 4 °C for egg quality determination. In the last but 3 days, 1 hen was randomly selected from each replicate. Excreta without feathers and feed were collected every day for three times using the total fecal collection method and the feed intake for the 3 days was recorded for apparent metabolism and microbial analysis.

### Laying performance

Egg production, total egg weight, and broken-egg production (including broken, porous, cracked, or thin-shelled eggs) per replicate were recorded daily to calculate laying rate and broken-soft egg ratio. Total feed consumption and the leftovers for each replicate was weighed every week to calculate laying rate, average egg weight, average monthly egg weight, broken-soft egg ratio, average daily feed intake (ADFI), and Feed-Egg ratio. Feed-Egg ratio was calculated as grams of feed consumed/egg weight for each replicate.

### Egg quality

Egg weight, eggshell strength, eggshell thickness, albumen height, yolk color and Haugh unit were measured using a digital egg tester (DET6500, NABEL, Japan). Egg length and egg width were measured by an outside micrometer for calculating shape index (length/width ratio).

### Serum parameters

Total protein (TP), alanine aminotransferase (ALT), aspartate aminotransferase (AST), alkaline phosphatase (ALP), creatinine (CREA), urea nitrogen (UREA), calcium (Ca), and phosphorus (P) level were determined using automatic biochemical analyzer (ADVIA 2400, Simens, Germany). The total antioxidant capacity (T-AOC), catalase (CAT), glutathione peroxidase (GSH-Px), total superoxide dismutase (T-SOD), and malondialdehyde (MDA) level were determined using colorimetric kits (Nanjing Jiancheng Bioengineering Institute, Nanjing, China) in accordance with the manufacturer’s instructions. IgG, IgA, and IgM were determined by Multiskan MK3 microplate reader (Thermo Fisher Scientific Company). Serum reproductive hormones estradiol (E2) and progesterone (PROG) were determined using kits produced by Beijing Sino-British Biotechnology Institute. And the specific determination was performed by Beijing Sino-British Biotechnology Institute (Beijing, China).

### Nutrient apparent metabolism

Fecal samples were mixed using the weighted average sampling method, and 20 ml of 10% HCl was added to each 100 g of fresh feces and immediately stored at 4°C. After all samples had been collected and mixed, 10% of the samples were dried to constant weight in an oven at 65 °C and moistened at room temperature for 24 h. The samples were crushed and passed through a standard 40 mesh sieve. Apparent digestibility of apparent metabolizable energy (AME), dry matter (Liu et al., 2018a), crude protein (CP), crude fiber (CF), crude fat (EE), calcium (Ca), and phosphorus (P) in the diet and excretion in feces and urine were determined using the ‘Feed Analysis and Feed Quality Detection Technology (4th Edition)’. The formula for calculating the apparent metabolism of nutrients is as follows:

AME (MJ / kg) = (total energy intake – total energy of feces and urine) / total energy intake of diet Nutrient apparentmetabolic rate = 1 − (indicator content in the diet / indicator content in the fecesand urine nutrient content in the fecesand urine nutrient content in the diet ) ×100%

### Digestive enzyme activity of jejunal and ileal mucosa

The activities of amylase, lipase, and chymotrypsin in the mucosa from the jejunum and ileum were determined by colorimetry using assay kits (Nanjing Jiancheng Bioengineering Institute of China, Nanjing, China).

### Morphological and microbial analysis

After a series of gradient alcohol dehydration, xylene transparency and paraffin embedding, paraffin sections of 5 μm were made by automatic rotary slicing machine, routine HE staining and neutral gum fixation. Finally, the histological changes of the intestinal tract were observed using a light microscopy. Six groups of discontinuous visual fields were randomly selected for each section, and 4 groups of data were counted for each visual field. Villus height (VH, the height from the tip of villus to the villus-crypt junction) and crypt depth (CD, the depth of invagination between adjacent villi) were measured using Image-ProPlis 6.0 software. VH to CD ratio (VCR) was calculated. The overall average was taken as the final measurement.

The numbers of *E. coli*, *Salmonella*, and *Lactobacillus* were enumerated using selective culture media, including MacConkey agar, Shigella agar, and Rogosa agar, respectively (Beijing Huaying Biotechnology, Beijing, China). Coated plates were cultured for 24 h at 37 °C. Following the incubation periods, the colonies of respective bacterium were counted and expressed as log10 CFU/g of fecal sample.

### Statistical analysis

Excel 2019 was used for preliminary statistics of the experimental data, and SPSS Statistics 27.0 statistical software was used for one-way ANOVA, with Duncan’s multiple range test for post hoc comparison. Significance was set at *P* < 0.05.

## Results

### Effects of OEA and CEP on laying performance

Table 2 presents the effects of feed additives supplement strategies on the laying performance of Dagu breeder hens. Dietary supplementation of OEA and CEP had no effect on the average egg weight and ADFI of laying hens (*P *> 0.05). Compared with the CON group, the LCO group supplementation significantly increased the laying rate by 3.24%, improved the feed-egg ratio by 4.75%, and boosted the monthly total egg weight by 5.19% (*P *< 0.05), while the LOE group showed significant improvements of 2.19%, 3.59%, and 3.05% in the laying rate, feed-egg ratio, and monthly total egg weight, respectively (*P *< 0.05). Additionally, the LCO group showed lower broken-soft egg ratio (85.19%) than the CON group (*P *< 0.05).

**Table 2.** Effect of feed additives supplement strategies on laying performance of Dagu breeder hens

Item	CON	LCO	LCE	LOE	SEM^*^	*P*-value
Laying rate, %	66.220^b^	68.367^a^	67.670^ab^	69.556^a^	0.401	0.017
Feed-egg ratio	3.551^a^	3.390^b^	3.428^ab^	3.308^b^	0.029	0.015
Average egg weight (g)	51.760	52.713	52.165	53.503	0.376	0.411
Monthly total egg weight (kg)	16.438^c^	17.291^ab^	16.940^bc^	17.860^a^	0.143	0.001
ADFI (g)	121.595	122.010	120.951	122.995	0.415	0.382
Broken-soft egg ratio	0.050^a^	0.027^b^	0.044^a^	0.037^ab^	0.003	0.035

Different letters in the same column indicate significant difference (*P *< 0.05). The same as in the following table. ADFI: average daily feed intake.

### Effects of OEA and CEP on egg quality

Table 3 presents the effects of feed additives supplement strategies on the egg quality of Dagu breeder hens. Dietary supplementation of OEA and CEP showed a significant enhancement in eggshell strength by 7.66% (*P *< 0.05). Additionally, the supplementation of OEA alone and in combination with CEP increased the Haugh unit by 1.60% and 2.01%, respectively (*P *< 0.05), whereas those of eggshell thickness, yolk color and eggshell index as well as albumen height were not affected (*P *> 0.05).

**Table 3.** Effect of feed additives supplement strategies on egg quality of Dagu breeder hens

Item	CON	LCO	LCE	LOE	SEM^*^	*P*-value
Eggshell strength (N)	55.775^b^	59.225^a^	58.023^a^	60.045^a^	0.462	0.002
Eggshell thickness (mm)	0.378	0.387	0.365	0.383	0.016	0.086
Yolk color	7.750	7.917	7.833	8.000	0.495	0.856
Eggshell index	1.320	1.325	1.335	1.340	0.005	0.552
Albumen height (mm)	6.275	6.321	6.297	6.370	0.026	0.620
Haugh unit	77.450^b^	78.690^a^	77.893^ab^	79.008^a^	0.266	0.031

Different letters in the same column indicate significant difference (P < 0.05).

### Effects of OEA and CEP on serum parameters

Table 4 presents the effects of feed additives supplement strategies on the serum biochemical parameters of Dagu breeder hens. The serum levels of TP and Ca was higher by 4.31% and 7.97% (*P *< 0.05) in LOE group than that in CON group, and the supplementation of OEA alone and in combination with CEP increased AST, CREA, and P by 9.48%, 4.35% and 12.37%, respectively (*P *< 0.05). In contrast, the serum levels of AST, CREA, and P in LCE group has an improvement trend but not significant (*P *> 0.05) compare with those in CON group.

**Table 4.** Effect of feed additives supplement strategies on serum biochemical parameters of Dagu breeder hens

Item	CON	LCO	LCE	LOE	SEM^*^	*P*-value
TP (g/L)	61.050^b^	62.950^ab^	61.967^ab^	63.683^a^	0.361	0.042
ALT (U/L)	33.843	33.098	33.126	31.148	0.504	0.276
AST(U/L)	188.667^a^	174.333^b^	178.667^ab^	172.333^b^	2.276	0.040
ALP (U/L)	232.333	226.167	231.000	224.000	2.120	0.485
CREA (μmol/L)	58.000^a^	56.000^bc^	57.583^ab^	55.583^c^	0.359	0.031
UREA (mmol/L)	5.372	5.258	5.312	5.333	0.027	0.555
Ca (mmol/L)	2.008^b^	2.097^ab^	2.067^ab^	2.168^a^	0.025	0.024
P (mmol/L)	1.698^c^	1.838^ab^	1.777^bc^	1.908^a^	0.024	0.006

Different letters in the same column indicate significant difference (P < 0.05). TP: total protein; ALT: alanine aminotransferase; AST: aspartate aminotransferase; ALP: alkaline phosphatase; CREA: creatinine; UREA: urea nitrogen; Ca: calcium; P: phosphorus

Table 5 presents the effects of feed additives supplement strategies on the serum antioxidant parameters of Dagu breeder hens. The supplementation with OEA + CEP in diet significantly improved the antioxidant capacities of T-AOC, GSH-Px and T-SOD on Dagu breeder hens by 7.02%, 11.31% and 5.02%, respectively (*P *< 0.05), and OEA also significantly increased the levels of T-AOC and GSH-Px by 6.66% and 7.97% (*P *< 0.05). Additionally, the MDA level was lower in LOE group than the other groups by 7.93% (*P *< 0.05).

**Table 5.** Effect of feed additives supplement strategies on serum antioxidant parameters of Dagu breeder hens

Item	CON	LCO	LCE	LOE	SEM^*^	*P*-value
T-AOC (U/ml)	6.155^b^	6.565^a^	6.323^ab^	6.587^a^	0.055	0.006
CAT (U/ml)	7.892	8.072	8.072	8.087	0.037	0.209
GSH-Px (U/ml)	1378.567^c^	1488.385^ab^	1449.076^bc^	1534.458^a^	13.776	<0.001
T-SOD (U/ml)	174.560^b^	177.096^b^	177.270^b^	183.315^a^	0.945	0.002
MDA (nmol/ml)	2.817^a^	2.698^ab^	2.770^ab^	2.610^b^	0.029	0.050

Different letters in the same column indicate significant difference (*P* < 0.05). T-AOC: total antioxidant capacity; CAT: catalase; GSH-Px: glutathione peroxidase; T-SOD: total superoxide dismutase; MDA: malondialdehyde

Table 6 presents the effects of feed additives supplement strategies on the serum immune parameters and reproductive hormones of Dagu breeder hens. Dietary supplementation of OEA alone and in combination with CEP increased in IgM levels by 4.26% and 5.54% (*P *< 0.05). Compared with CON group, OEA +CEP increased IgG levels by 6.27% (*P *< 0.05). No significant difference was found in IgA in the serum (*P *> 0.05). Additionally, increases of 3.75% and 5.81% in the E2 level were observed in the LCO and LOE groups (*P *< 0.05).

**Table 6.** Effect of feed additives supplement strategies on serum immune parameters and reproductive hormones of Dagu breeder hens

Item	CON	LCO	LCE	LOE	SEM^*^	*P*-value
IgG (μg/mL)	2338.426^b^	2415.540^ab^	2389.037^b^	2484.936^a^	19.292	0.044
IgA (μg/mL)	367.826	374.038	371.878	372.392	2.084	0.777
IgM (μg/mL)	823.567^b^	858.663^a^	836.213^b^	869.195^a^	5.585	0.007
PROG (ng/mL)	19.491	21.655	19.588	21.367	0.472	0.224
E2 (pg/mL)	263.375^b^	273.250^ab^	265.270^b^	278.667^a^	2.246	0.044

Different letters in the same column indicate significant difference (*P* < 0.05). PROG: progesterone; E2: estradiol

### Effects of OEA and CEP on nutrient apparent metabolism

Table 7 presents the effects of feed additives supplement strategies on the apparent metabolism of Dagu breeder hens. All groups showed improved AME value, DM, CP, and Ca levels following dietary supplementation, with the most pronounced effect seen in the combination group, which exhibited increases of 4.33%, 4.15%, 4.92%, and 4.83%, respectively (*P *< 0.05), while did not affect the CF and EE levels of laying hens (*P *> 0.05). Additionally, LOE group showed a significant increase in P level by 2.53% (*P *< 0.05), while LCO and LCE groups had an improvement trend but not significant (*P *> 0.05) compared with CON group.

**Table 7.** Effect of feed additives supplement strategies on apparent metabolism of Dagu breeder hen

Item	CON	LCO	LCE	LOE	SEM^*^	*P*-value
AME/(MJ/kg)	76.810^b^	79.527^a^	79.428^a^	80.138^a^	0.342	<0.001
DM, %	68.118^b^	70.187^a^	70.358^a^	70.946^a^	0.358	0.019
CP, %	59.501^b^	61.425^a^	61.768^a^	62.427^a^	0.326	0.004
CF, %	23.807	25.091	24.057	25.635	0.346	0.202
EE, %	71.567	72.812	72.595	73.743	0.420	0.353
Ca, %	49.050^b^	51.202^a^	50.773^a^	51.420^a^	0.325	0.029
P, %	49.948^b^	51.677^a^	51.213^ab^	51.827^a^	0.270	0.045

Different letters in the same column indicate significant difference (*P* < 0.05). AME: apparent metabolizable energy; DM: dry matter; CP: crude protein; CF: crude fiber; EE: crude fat; Ca: calcium; P: phosphorus

### Effects of OEA and CEP on intestinal homeostasis

Table 8 presents the effects of feed additives supplement strategies on the intestinal digestive enzyme activity of Dagu breeder hens. The LCO, LCE, and LOE groups enhanced lipase by 2.64%, 11.62% and 12.86%, respectively (*P *< 0.05). Additionally, LOE group showed higher α-amylase (9.23%) and trypsin (9.99%) than the CON group (*P *< 0.05).

**Table 8.** Effect of feed additives supplement strategies on intestinal digestive enzyme activity of Dagu breeder hens

Item	CON	LCO	LCE	LOE	SEM^*^	*P*-value
α-amylase (U/mg prot)	231.015^b^	233.786^ab^	240.179^ab^	252.332^a^	2.626	0.010
Lipase (U/mg prot)	2.177^b^	2.345^a^	2.430^a^	2.457^a^	0.038	0.024
Trypsin (U/mg prot)	399.967^b^	406.382^ab^	415.951^ab^	439.942^a^	4.722	0.007

Different letters in the same column indicate significant difference (*P* < 0.05).

Table 9 presents the effects of feed additives supplement strategies on the intestinal morphology of Dagu breeder hens. Dietary supplementation of OEA and CEP did not affect the VH and CD of jejunum as well as the VH of ileum (*P *> 0.05), while a significant enhancement was found in the VCR of jejunum (4.54%) in LOE group than the CON group (*P *< 0.05). The CD of ileum was lower in LOE group than that in CON (6.01%) and LCE (4.80%) groups (*P *< 0.05). The LCO and LOE groups showed higher the VCR of ileum than the CON and LCE groups (*P *> 0.05).

**Table 9.** Effect of feed additives supplement strategies on intestinal morphology of Dagu breeder hens

Item		CON	LCO	LCE	LOE	SEM^*^	*P*-value
Jejunum	VH (μm)	1188.500	1211.167	1202.000	1230.000	6.727	0.168
	CD (μm)	176.000	174.667	173.833	174.167	1.056	0.908
	VCR	6.755^b^	6.938^ab^	6.917^ab^	7.062^a^	0.037	0.020
Ileum	VH (μm)	972.167	976.333	982.500	987.167	3.037	0.327
	CD (μm)	135.000^a^	131.000^ab^	134.833^a^	129.333^b^	0.814	0.017
	VCR	7.205^c^	7.452^b^	7.288^bc^	7.638^a^	0.044	<0.001

Different letters in the same column indicate significant difference (*P* < 0.05). VH: villus height; CD: crypt depth; VCR: villus height-to-crypt depth ratio

Table 10 presents the effects of feed additives supplement strategies on bacterial flora in feces of Dagu breeder hens. The numbers of *E. coli, Salmonella*, and *Lactobacillus* in excreta were determined. OEA significantly decreased the number of *Escherichia* in excreta by 89.22% (*P *< 0.05), and OEA+ CEP also showed positive influence on *Escherichia* (*P *> 0.05). However, OEA and CEP had no effect on the numbers of *Salmonella, and Lactobacillus* in the feces of Dagu breeder hens.

**Table 10.** Effect of feed additives supplement strategies on bacterial flora in feces of Dagu breeder hens

Item	CON	LCO	LCE	LOE	SEM^*^	*P*-value
*Escherichia coli*/lg (CFU/g)	3.460^a^	3.072^b^	3.428^a^	3.087^ab^	0.068	0.031
*Salmonella*/lg (CFU/g)	2.552	1.842	2.263	2.183	0.101	0.056
*Lactobacillus*/lg (CFU/g)	6.667	7.180	6.756	6.940	0.104	0.344

Different letters in the same column indicate significant difference (*P* < 0.05).

## Discussion

The main active ingredients of OEA are carvacrol and thymol with LA. It is worth mentioning that thymol and carvacrol in OEO exhibit to have antibacterial and anti-inflammatory properties and enhance nutrient utilization and improve gut health. It has been reported that OEO can improve the feed conversion rate of broilers (Pirgozliev et al., 2019) and laying hens (Ding et al., 2017) when used as a growth promoter. Studies have found that adding 200 mg/kg OEO to the diet significantly increased egg weight of laying hens (Gul et al., 2019). In addition, as a feed additive for broilers, LA improves feed conversion due to its unique nutritional and antibacterial effects (Jadhav et al., 2021). Some previous studies have observed that the addition of xylanase, protease and amylase has a small effect on laying rate and egg weight in laying hens (Al-Saffar et al., 2013). Those above are consistent with our finding. Córdova-Noboa et al. (2020) found that amylase supplementation increased nutrient digestibility and energy utilization, consequently, the usage of an exogenous amylase was effective on improving the live performance of broilers. Wan et al. (2023) reported that proteinase and cellulase enzymes supplementing a flaxseed diet can significantly increase egg weight, while using multiple carbohydrase and protease enzymes can enhance egg production. The current study showed that OEA and OEA + CEP increased laying rate, monthly total egg weight and decreased Feed–Egg ratio in Dagu hens, while CEP had a slight improvement on laying rate and Feed–Egg ratio. These results align with previous research that the supplementation with mixed organic acids and essential oils could increase laying rate and decreased the feed conversion ratio (Wang et al., 2019). The improved performance may be reduced chymus viscosity as a result of hydrolysis of soluble NSP and the release of nutrients leading to improved nutrient and energy utilization, simultaneously, the effective components of OEA and CEP may stimulate the secretion of digestive enzymes, thereby improving production performance parameters of birds. Whereas OEA and CEP had no significant influence on ADFI, which may be related to the spicy taste, type and dose of OEA and the offset of CEP. In addition, OEA reduced the broken-soft egg ratio, which may be related to the improvement of eggshell strength. Surprisingly, the combination of OEA and CEP improved laying rate, egg weight and Feed–Egg ratio better than the addition of OEA alone. We speculate that there is a complementary effect between the various enzymes in CEP and the active components of OEA, which can effectively maintain body growth and high laying rate, and the synergistic mechanism is worthy of further investigation.

Egg quality is one of the most important factors affecting the economic benefits of egg industry. In previous studies, dietary OEO supplementation improved the eggshell thickness of late-phase laying hens (Feng et al., 2021). On the contrary, it was reported that supplementation of the layer diet with EO had no significant effects on Haugh Unit, yolk color, eggshell weight and eggshell thickness (Torki et al., 2021). Additionally, Studies have shown that the effect of enzymes on mineral indicators in the serum of laying hens is often manifested in the bones (Olgun et al., 2018). The results of our study indicated that dietary supplementation of OEA and CEP alone or in combination contributed to an improvement in eggshell strength level, and showing a trend of increased eggshell thickness in Dagu breeder hens, which may be related with deposition of calcium and phosphorus based on the addition of OEA and CEP. OEA and CEP synergistically improved eggshell quality the improvement in eggshell quality. It has been reported that EO can improve uterine health and provide an appropriate site for eggshell calcification, thereby increasing eggshell weight and thickness (Abdel-Wareth and Lohakare, 2020). Our results prove this, as evidenced by OEA increasing the apparent metabolism of Ca and P in Dagu hens. In addition, some previous studies have shown that OEA can significantly increase the Haugh unit (Özek, 2012), which is consistent with our study.

To investigate the metabolic processes involved in OEA, CEP and OEA+CEP promoting the laying performance of Dagu breeder hens, we analyzed serum biochemical parameters associated with nutrition metabolism. Serum parameters analysis can reflect the health status of animals, including changes caused by internal and external interference factors (Liu et al., 2016). Serum protein is mainly synthesized in the liver and its concentration reflects the function of liver cells. Carvacrol (one of the effective constituents of OEA) is known to have anti-inflammatory, anti-hepatotoxicity and hepatoprotective activities (Baser, 2008). Studies have found that that the feeding of 150 mg/kg OEO increased serum total protein levels (Migliorini et al., 2019). Moreover, most of the LA ingested into the body is directly transported to the liver and converted into a direct form of energy in the liver(Dayrit, 2015). We found that the addition of 200 mg/kg of OEA and 200 mg/kg of CEP to the diet can increase the levels of TP, which showed the synergistic effect of the OEA and CEP. Moreover, the activity levels of ALT and AST can be used to evaluate the liver toxicity (Paul et al., 2016). The addition of OEO has been reported to reduce levels of ALT and AST (Abo Ghanima et al., 2020), while others have been reported to have no effect on ALT and AST levels (Bölükbaşi̇ et al., 2010; Lokaewmanee et al., 2014). In our finding, adding 200 mg/kg OEA to the diet reduced AST levels, the level of ALT has a slight decreasing trend. OEO may improve renal dysfunction in neonatal diarrhea calves by reducing creatinine (Katsoulos et al., 2022), which aligns with our results. Indeed, it was surprising to find that the combined use of OEA and CEP had a beneficial effect on hepatic and renal anabolism in Dagu hens, with a significant reduction in AST and CREA levels. Furthermore, studies have shown that feeding LA-rich coconut oil can significantly increase the solubility of Ca and P in serum and affect their metabolism in vivo (Demirci et al., 2023). Hereby, dietary supplementation of OEA + CEP increased Ca level in the serum, while dietary supplementation of OEA as well as OEA + CEP in combination increased P level. The content of Ca and P in serum showed a potential positive effect on their metabolism, even eggshell quality.

Owing to OEA is widely known for its antimicrobial and antioxidant effects, which improve birds gut health and enhance nutrient absorption, and it is commonly used as additive in diet (Hafeez et al., 2016). Studies have shown that the addition of OEO to broiler diets significantly increased the levels of GSH-Px, SOD and T-AOC in serum(L. Y. Zhang et al., 2021). In addition, it has been reported that dietary with 200 mg/kg OEO showed lower MDA levels than more doses of OEO(Gul et al., 2019), which aligns with our results. A study has shown 350 and 500 mg/kg of lauric acid monoglyceride and cinnamaldehyde complex plant essential oils can improve the body’s antioxidant capacity (Zheng et al., 2023). Early studies demonstrated that xylanase increased T-AOC, SOD and GSH-Px levels, and reduced MDA levels of broilers (Zhang et al., 2018), and compound enzymes (containing amylase, protease, xylanase and β-glucanase) could increase serum SOD content, and decreased MDA content in weaned piglets (Long et al., 2021). We found that supplementation of OEA and OEA + CEP in the diet significantly increased T-AOC and GSH-Px levels. Consistent with our study results, Bozkurt et al. (2016), reported that carvacrol and thymol have exhibited considerable antioxidant activity, OEA also have been reported to improve antioxidant stability (Liu et al., 2018b; Anuar et al., 2023), to increase serum SOD and GPX activities (Liu et al., 2022). Zhou et al. (2023) reported that feeding with compound enzyme preparation for 56 days significantly improved the productive performance but did not affect the antioxidative capacity and immunity of goats, it can be obtained a similar phenomenon in biochemical experiments with Dagu hens. Notably, the reduction of MDA in accordance with the increase of SOD activity showed the synergistic effect of OEA and CEP on the antioxidation of laying hens which may prove that we made an appropriate ratio of OEA and CEP.

EO compounds can stimulate protein synthesis and the immune system and protect cells from the effects of oxidative processes. The enhancement of immunoglobulins observed on the laying hens fed with EO may be related to improved immune responses (Mousavi et al., 2018). Studies have reported that the addition of OEO to the broiler diet increased IgG but not significantly (Alp et al., 2012). Mixed organic acid supplementation increased the serum IgA level in broilers (Ma et al., 2021). Supplemental OEA improved feed conversion efficiency and cecal *E. coli* population, enhanced serum IgG concentration (Pham et al., 2020), and increased levels of IgA and IgM, which contribute to the enhancement of immune status (Dong et al., 2024). However, another study showed that OEA had no difference in the secretory IgA content of the jejunum (Zhang et al., 2019). Previous study showed that compound enzymes could increase serum IgA, IgG, and IgM in weaned piglets(Long et al., 2021). In our study, the addition of OEA and OEA+ CEP significantly increased the IgM level. Compared with the CON group, the addition of OEA and CEP in combination significantly increased the IgG level, likely through complementary mechanisms: CEP improves nutrient availability, supplying raw materials for antibody synthesis. OEA helps direct these resources toward immune function by reducing oxidative stress and supporting metabolic health. The pronounced increase in IgM suggests that OEA and CEP work together to strengthen innate and mucosal immunity more effectively than either alone. In addition, a recent study found that there was a slight increase in the number of mature oocytes in the ovaries of female carp fed OEO (Yigit et al., 2024). Ovarian reproductive function and egg quality may be improved by stimulating ovarian estrogen secretion. Our results showed dietary supplementation of OEA + CEP significantly increased the level of serum E2. No previous research was published concerning reproductive hormone of laying hens fed with the diet supplemented with OEA and CEP. Some scholars found in human follicles that the higher the concentration of E2 the better the quality of follicles cultured in vitro (von Wolff et al., 2021). Whether OEA and CEP could improve the follicular development of layers by increasing the concentration of PROG and E2 remains to be further studied.

Given the above differences in laying performance, egg quality and serum parameters, this study further investigated the effects of OEA and CEP on apparent metabolic rate and intestinal morphology in Dagu breeder hens. Studies have shown that dietary with OEA 300 mg/kg may maintain intestinal tract morphology and promote digestive and absorptive capacities (Wang et al., 2019), and 100 or 200 mg/kg EOs can improve the digestibility of dry matter, organic matter and crude protein (Arslan et al., 2022). OEA have been reported to improve mineral utilization, act as an energy source, and promote endogenous enzyme secretion (Liu et al., 2018).

Study demonstrates multi-enzyme or xylanase supplementation could increase the nutrient digestibility (Iqbal et al., 2019). Previous studies have shown that supplementation of enzymes (a mixture of mannanase, α-galactosidase and protease) in the diet of laying hens can significantly improve AME (Chong et al., 2008). Similarly, amylase has a significant effect on the AME of broilers (Kaczmarek et al., 2014). After supplementation of xylanase, the percentage of metabolic energy coefficient of dietary crude protein was higher (de Souza et al., 2012). Our results show that the addition of OEA and CEP improved the utilization of AME, DM, CP and Ca levels, which is similar to previous results. Improved digestion may be due to OEA increasing the digestive enzyme activities of both pancreas and intestinal mucosa which could be another explanation of better nutrient digestibility, and the addition of mixed enzymes to the diet can improve the breakdown of cell wall polysaccharides in plant raw materials, thereby improving nutrient utilization by chickens (Khempaka et al., 2018). We believe that due to the characteristics of roughage resistance of Dagu hens, it shows a higher digestion and utilization rate of crude protein, and also improves the activity of α-amylase in the intestine, which explains our results. In addition, OEA also increased the body’s metabolism of P, which may be related to the increase in egg production and the nutritional supply of eggshells. We were surprised to find the combined use of OEA and CEP enhancing the body’s immunity and improving the production performance.

Improved digestibility can be predicted to occur with higher digestive enzyme activity. It has been reported that EO could stimulate digestive secretions such as bile acids, gastric and digestive enzymes (Platel and Srinivasan, 2004). Likewise, previous studies have described an increase in lipase, trypsin and amylase activity following the addition of a mixture of carvacrol and thymol to the broiler diet (Meligy et al., 2023). Ma et al. (2021) reported that mixed organic acid decreased the pH value of the duodenum and enhanced the amylase activity of the pancreas, nevertheless had no impact on the trypsin, lipase, and chymotrypsin activities. It has been reported that α-amylase preparation increased intestinal trypsin activity in broiler diets (Jiang et al., 2008). Our results are similar owing to CEP contains amylase ≥ 600 μ/g. The dietary supplementation of OEA and CEP in combination improved α-amylase, lipase, and trypsin, while the dietary supplementation of OEA and CEP alone can also increase lipase. The results showed that OEA and CEP had an interaction effect on the digestion and utilization of nutrients in Dagu hens. The apparent synergistic effect between OEA and CEP on nutrient metabolism and digestive enzyme activity observed in this study can be mechanistically explained by a potential sequential mode of action. Firstly, CEP (especially NSP-degrading enzymes) breaks down plant cell walls, releasing encapsulated nutrients and increasing their accessibility. Subsequently, the oregano essential oil and lauric acid blend (OEA) may function as a secondary enhancer in two ways by: 1) inhibiting detrimental gut bacteria that compete for nutrients, and 2) stimulating the secretion of the host’s endogenous digestive enzymes and improving intestinal absorption. In essence, CEP “unlocks” the nutrients from the feed matrix, while OEA “safeguards” these nutrients from microbial competitors and “potentiates” the host’s digestive and absorptive functions.

Gut morphology is one of important indirect markers for assessing intestinal health, recovery and functionality. In general, it is believed that increased intestinal mucosal thickness, especially villus height, is associated with increased digestive and absorptive function of the small intestine due to increased absorption surface area (Galli et al., 2020). A linear increasing in ileal villus height of early-phase period laying hens fed OEA from 150 to 300 mg/kg was observed (Wang et al., 2019), and also a same positive effect on the intestinal morphology was demonstrated (Kaya et al., 2015). We observed that dietary supplementation with OEA significantly increased the VCR of ileum, which is consistent with previous findings (Du et al., 2016; Pham et al., 2020). The enhancement of ileal VCR associated with the supplementation of xylanase in laying hens diets (de Souza et al., 2014). Our current results showed that CEP can slightly increase the VCR of jejunum and ileum. When OEA and CEP are used in combination, they showed a significant complementary effect to improve the VCR of the jejunum and ileum as well as the CD of ileum. In an early broiler study, it was found that the ileum villus height increased at a high dose of xylanase (90,000 U/g), indicating that the effect of xylanase on intestinal morphology was dose-dependent (Van Hoeck et al., 2021). We speculate that CEP contains other enzymes in addition to xylanase, which has the same effect as high-dose xylanase. In conclusion, OEA and CEP are used in combination can improve the intestinal morphology, enhance digestion and nutrient absorption, and achieve better production efficiency.

Some studies have demonstrated that OEO and LA could maintain broiler chickens intestinal health by inhibiting or killing Gram-negative and Gram-positive bacteria including *Salmonella*, *E. coli*, and *Clostridium perfringens*, without harming beneficial bacteria like *Lactobacillus* spp. (Altieri et al., 2009; Stefanello et al., 2019; Rolinec et al., 2020). Remarkable growth inhibition of *E. coli* and a slight increasing numbers of *Lactobacillus* were observed after supplementation with OEA in our study. Consistent with our study, some authors have reported that the addition of organic acid resulted in a more appropriate environment in the caecum for beneficial microorganisms (Dauksiene et al., 2021), and OEA had a positive impact on the gut microflora of the ileum (Adaszynska-Skwirzynska and Szczerbinska, 2019), and suggested that supplemental OEA could increase the proportion of *Lactobacillus spp.* in chickens (Dai et al., 2020). It is important to note that the 40-day duration of the feeding trial, while sufficient to evaluate initial efficacy, is a limitation of this study for assessing the long-term persistency of the observed effects on laying performance and overall hen health. Future studies employing longer experimental periods (e.g., a full laying cycle for ≥8 weeks) are warranted to confirm the sustainability of these benefits. Next, it is necessary for us to further study and explore whether the interaction mechanism between the two feed additives and the more ideal ratio in order to extend the productivity cycle of Dagu breeding hens.

## Conclusion

In summary, dietary OEA and CEP, individually or combined, enhanced egg production, shell quality, antioxidant and immune capacity, and nutrient utilization in Dagu breeder hens. Nevertheless, the synergistic mechanisms of OEA and CEP and their long-term effects on hen health and laying persistence warrant further investigation. Notably, OEA showed marked antioxidant benefits, and its combination with CEP highlights promising potential for the poultry industry.

## Data Availability

The raw data supporting the conclusions of this manuscript will be made available by the authors, without undue reservation, to any qualified researcher.

## Abbreviations:

ADFI: Average Daily Feed Intake
ALP: Alkaline Phosphatase
ALT: Alanine Aminotransferase
AME: Apparent Metabolizable Energy
AST: Aspartate Aminotransferase
CAT: Catalase
CD: Crypt Depth
CEP: Compound Enzyme Preparation
CF: Crude Fiber
CP: Crude Protein
CREA: Creatinine
E2: Estradiol
EE: Crude Fat
EO: Essential Oil
GSH-Px: Glutathione Peroxidase
LA: Lauric Acid
MDA: Malondialdehyde
OEA: Oregano Essential Oil And Lauric Acid
PROG: Progesterone
T-AOC: Total Antioxidant Capacity
TP: Total Protein
T-SOD: Total Superoxide Dismutase
UREA: Urea Nitrogen
VCR: Villus Height-to-Crypt Depth Ratio
VH: Villus Height
